# Cervical cancer screening behaviours and challenges: a sub-Saharan Africa perspective

**DOI:** 10.11604/pamj.2020.36.97.19071

**Published:** 2020-06-16

**Authors:** Judith Anaman-Torgbor, Seth Kwadjo Angmorterh, Dzifa Dordunoo, Eric Kwasi Ofori

**Affiliations:** 1 Department of Public Health Nursing, School of Nursing and Midwifery, University of Health and Allied Sciences (UHAS), PMB 31, Ho-Volta Region, Ghana,; 2 Department of Medical Imaging, School of Allied Health Sciences, University of Health and Allied Sciences (UHAS), PMB 31, Ho-Volta, Ghana,; 3 School of Nursing, University of Victoria, HSD 460 P O BOX 1700 STN CSC Victoria BC V8W 2Y, Canada

**Keywords:** Cervical cancer, public health, cancer

## Abstract

Cervical cancer may be fatal to women if not identified and treated early. The importance of organised cervical screening has been felt in many developed countries. However, the majority of women in developing countries may be under-screened or may have never been screened because many developing countries have not developed a national cervical cancer prevention program accessible to all women due to reasons such as competing funding priorities, low prioritization of cervical cancer and cultural practices across Africa. It is important that these factors are adequately addressed to improve access to regular cervical screening services and ultimately help curb the incidence and impact of cervical cancer on women in sub-Saharan Africa.

## Commentary

Cervical cancer is a significant public health issue especially in developing countries. This is because the incidence of invasive cervical cancer in sub-Saharan Africa is one of the highest in the world, with about 35 new cases per 100,000 women and 23 per 100,000 women dying from the disease each year [1]. Cervical cancer as documented is preventable and curable when detected early in its pre-cancerous stages. That is regular screening of the cervix with a high-quality Pap smear helps in identifying pre-cancerous changes in the cells of the cervix and treated before they progress to cancer [2]. However, resources for cervical screening are lacking in developing countries and access to cervical cancer screening is also low. As a result, few women in sub-Saharan Africa have used cervical cancer screening services. In Ghana for instance only very few women reported ever having a pelvic examination. In most developing countries cervical screening starts as opportunistic screening initiated by health professionals or by the patients themselves. The unavailability of regular cervical screening programs in developing countries is mostly due to competing funding priorities, low prioritisation of screening services and few trained and skilled healthcare service providers available to perform or implement screening programs effectively. Despite the number of public awareness and health promotion activities to encourage the public, cervical screening uptake has remained very low in the sub-region. This commentary aims to provide an overview of cervical cancer prevention practices and highlight factors influencing African women´s behaviour towards regular cervical cancer screening services in the sub-Saharan African region.

**Cervical cancer prevention:** there are over 200 types of HPVs and about 40 of them are known to affect the genital tract and HPV types 6, 11, 16, 18, 31, 33, 35, 45 and 58 have been associated with cervical cancer [3]. Infections with one or more of the many types of HPV are extremely common and most HPV infections are asymptomatic and often cleared by the immune system. However, the infection may persist in some women and lead to changes in the cells of the cervix and ultimately to cervical cancer. Cervical cancer is one of the most preventable human cancers due to its slow disease progression and given the unique association of specific HPV types with cervical cancer, preventive strategies such as vaccination and/or regular screening targeted at specific sub-populations are highly effective. Primary prevention strategy for cervical cancer aims to vaccinate both girls and boys against the HPV while secondary prevention targets screening and treating women 30 years or older. Screening, is defined as “*the systematic application of a test or inquiry, to identify individuals at risk of a specific disorder to benefit from further investigation or direct preventive action, among persons who have not sought medical attention on an account of that disorder*” [4]. Recommended cervical cancer screening tests include cervical cytology, HPV DNA testing, visual inspection, colposcopy, and cervicography. Other emerging techniques include computer-assisted cytological interpretation of cervical smears, and physical real-time detection of molecular surrogate makers of cancer progression. Tertiary prevention strategies aim to treat and palliate invasive cancer at any age and as most adult women in low-income countries in sub-Saharan Africa have not had the benefit from the recently developed HPV vaccines and are therefore at greater risk of exposure to HPV, it is important for them to have regular cervical screening for early identification of pre-cancerous changes is paramount.

**Impact of regular cervical screening:** regular cervical screening programs are common in developed countries and there are evidence showing that screening programs produce positive results. A single occurrence of cervical cancer screening programmes and services reduce a woman´s risk of cervical cancer for the next 5 years by 50% [5]. In Australia for instance, the introduction of regular cervical cancer screening programme resulted in a drastic reduction in incidence rate, from 13.3 per 100,000 in 1991 to about 7 per 100,000 between 2001 and 2008. Similarly, in England, there was a reduction in stage 1A and stage 3 or worse cervical cancer with 70% reduction of cervical cancer deaths among women of all ages [6]. Compare to women with no or minimal screening participation those who undergo regular screening are 82% less likely to get cervical cancer. Although cervical screening programs have been successful in curbing the incidence of cervical cancer in developed countries very few women in sub-Saharan Africa have used screening services. Many developing countries lack a national or regular cervical cancer prevention program accessible to all women. This may largely be due to reasons such as competing funding priorities, low prioritization of cervical cancer, and few trained and skilled healthcare service providers able to implement screening programs effectively. The few available cervical screening services in sub-Saharan Africa are usually found in secondary and tertiary healthcare facilities located in urban areas. Ghana for example does not have a national or regular cervical screening program, but instead, opportunistic screening occurs when medical doctors recommend Pap smear testing or Visual Inspection with Acetic-acid (VIA) in response to patient´s symptomatology. While there have been a number of public awareness and health promotion activities to encourage the public to be screened in Ghana, screening uptake has remained very low.

**Health seeking behaviour: African perspectives:** in many African societies the ascribed causes of diseases are diverse, ranging from the natural to the metaphysical (supernatural) [7]. The “natural” is when the explanation of the cause of a disease is similar to the scientific paradigm, whereas the metaphysical is when the source of a disease is attributed to spirits or ancestral mediations. In most traditional settings in Africa, it is commonly believed that the primary origin of illness is supernatural and that bacteria, viruses, and parasites are secondary causes of illness [7]. Therefore, both traditional and western medicine are consulted in traditional African settings to understand and explain the cause of diseases and the process of seeking treatment could largely be determined by the knowledge and understanding of the causation factor(s). Where the source of a disease or the causation factor is unknown, the disease is attributed to supernatural causes, and cures or explanations are sought from fetish shrines, diviners or spiritualists, because it is believed western medicine can neither provide explanation nor cure [7]. The belief is that a disease with unknown aetiology is primarily the result of offences against one´s own spirit, against an ancestral spirit, against the gods of the land, or due to the neglect or omission of a duty on the part of the victim.

**Socio-cultural constraints that impact on African women´s health-seeking behaviour:** cultural diversity is a known characteristic of the African continent. Although cultural characteristics vary both across and within African countries, there are similarities in cultural practices across Africa that may impact on the health status and health-seeking behaviours of African women. For instance, patriarchal culture: a system of social stratification that defines the role of men and women in relationships is a major feature in most traditional societies in Africa. Patriarchal culture provides material advantage to men and places severe constraints on women [8]. This creates social barriers for women, such as inequalities in marriage, education and employment. In social life, the interpersonal relations in families, schools, churches and workplaces are largely regulated by the dominance of males. In a traditional African society, the gender norms make men the primary authority in sex and reproductive health decision making while women are in a subordinate position with limited control over sexual matters, such as the ability to negotiate safe sex. The double standards on sexuality deny women the ability to refuse sex or negotiate condom use, while at the same time African men are allowed to have multiple sexual partners. As a result, women may not be able to protect themselves against sexual infections including HPV infection and unwanted pregnancies.

The socio-cultural differences between men and women in the African context can have detrimental effect on a woman´s health. Traditionally, an African man can have more than one wife if his wealth permits. Urbanisation and modernisation though has influenced this cultural norm and has increased the chance of a man having other sexual partners as opposed to wives. Although women may be aware that their partners have other multiple sexual partners, they often feel powerless to protest, and find it much harder to change their circumstances than to just accept their fate. The social and family role of many African women is to be a housewife, a mother and a caregiver fulfilling family and gender obligations through pregnancy and childbearing. In addition to suffering from inequalities in marriage and sexual relations, most women have less access to education, employment and health information and are economically dependent on their male partners [9]. Depending on the man for financial or economic support is a significant obstacle to safer-sexual decision-making, as the fear of losing this support may pressure women into having unprotected sex, thus putting them at increased risk of contracting sexually transmitted infections such as HPV.

These socio-cultural factors can prevent open discussions about sexual health or sex education, which reduces the opportunities for women to learn about sexual health issues. Knowledge about the cause of diseases is central to both modern and traditional medicine systems. This is because health-seeking behaviour is, to a very large extent, dependent upon the understanding and interpretation of the cause of illness. For instance knowledge about sexual health issues and condom use has been reported to be poor among African immigrant women living in Australia [10]. This could be associated with the socio-cultural reasons discussed above. It is therefore important for all women to be educated about sexual health and empowered to participate in regular cervical screen to safeguard their sexual and reproductive health.

## Conclusion

Cervical cancer is a significant public health issue worldwide because it has significant health implications on patients and healthcare providers. Although developed countries have national cervical cancer screening programmes to curb the incidence of cervical cancer, these programmes are not routinely available in developing countries including the sub-Saharan Africa. As a result, the majority of women in developing regions may be under-screened or have never been screened though affordable life-saving interventions are available to lessen the burden of cervical cancer. Proven intervention -Visual inspection with acetic acid (VIA) for instance is a simple, inexpensive screening test that can be combined with cryotherapy for early cervical lesions. Nonetheless, it has been demonstrated that testing the effectiveness of a scientifically proven intervention in developing world has come with challenges. This commentary highlighted two main factors influencing African women´s health seeking behaviour towards regular cervical cancer screening services and these factors increase the risk of cervical cancer incidence among women in traditional African settings. It is believed that highlighting of these factors will serves as a call to action by African health ministries and departments regarding cervical screening activities in the sub-Saharan African region.
